# Prevalence and factors associated with alcohol and drug-related disorders in prison: a French national study

**DOI:** 10.1186/1747-597X-2-1

**Published:** 2007-01-04

**Authors:** Michael Lukasiewicz, Bruno Falissard, Laurent Michel, Xavier Neveu, Michel Reynaud, Isabelle Gasquet

**Affiliations:** 1 INSERM, U669, Paris, F-75014 France ; Univ Paris-sud 11, Le Kremlin Bicêtre, F-94000 France; Univ Paris 5, Paris, F-75015 France; AP-HP, Villejuif, F-94804, France; 2 Centre de Recherche et de Traitement des Addictions., Assistance Publique-Hopitaux de Paris, Hôpital Paul Brousse, 14 avenue PV Couturier, Villejuif 94804, France; 3 Direction de la politique Médicale, Assistance Publique – Hôpitaux de Paris, 3 avenue Victoria, 75184 Paris cedex 4, France

## Abstract

**Background:**

Most studies measuring substance-use disorders in prisons focus on incoming or on remand prisoners and are generally restricted to drugs. However, there is evidence that substance use initiation or continuation occurs in prison, and that alcohol use is common. The aim of this study is 1) to assess prevalence of both drug and alcohol abuse and dependence (DAD/AAD) in a national randomised cohort of French prisoners, short or long-term sentence 2) to assess the risk factors associated with DAD/AAD in prison. a stratified random strategy was used to select 1) 23 prisons among the different types of prison 2) 998 prisoners. Diagnoses were assessed according to a standardized procedure, each prisoner being assessed by two psychiatrists, one junior, using a structured interview (MINI 5 plus), and one senior, completing the procedure with an open clinical interview. At the end of the interview the clinicians met and agreed on a list of diagnoses. Cloninger's Temperament and Character Inventory (TCI) was also used.

**Results:**

More than a third of prisoners presented either AAD or DAD in the last 12 months. Cannabis was the most frequent drug and just under a fifth of prisoners had AAD. AAD and DAD were clearly different for the following: socio-demographic variables, childhood history, imprisonment characteristics, psychiatric comorbidity and Cloninger's TCI. Profiles of AAD in prison are similar to type II alcoholism.

**Conclusion:**

Regular screening of AAD/DAD in prison, and specific treatment programmes taking into account differences between prisoners with an AAD and prisoners with a DAD should be a public health priority in prison

## Background

Drug users are over-represented in prisons in Europe. Nevertheless, data on patterns of drug use among prisoners are rare and difficult to interpret: Indeed, a large part comes from non-controlled or local studies, using different data collection methods. Furthermore, the fear of confidentiality breaches may bias prisoners' answers. Finally, systematic screening is unusual in prison setting, concerning less than 60% of incoming prisoners in France [1-4]. Lifetime prevalences across Europe for regular drug use prior to imprisonment vary widely, from 8% to 73% [5]. In France, in 2003, just over 30% reported alcohol abuse and a third regular drug use in the past 12 months [6].

### Estimates of abuse/dependence prevalence in prisons

Few studies have focused on standardized diagnoses of alcohol or drug abuse/dependence (AAD and DAD) among prisoners, and most were limited to incoming or remanded prisoners: estimates ranging from 25% to 74% for DAD and from 21% to 50% for AAD have been reported [7-13]. In contrast, with the notable exception of a national British study [14-17], few national studies have targeted the general prisoner population. However, substance misuse while in prison is not uncommon, whether ongoing substance misuse or first consumption: estimates ranges from 22% to 86%, cannabis being the most frequently reported illicit drug used (8–60%), and injecting being reported by 0–28% of inmates [5,18]. A British survey found that 60% of heroin users reported use in prison and more than 25% initiated use in prison [17]. AAD/DAD prevalence among all prisoners, not solely incoming/remanded prisoners, is still largely unknown in France.

### Studies on factors associated with substance use in prison

A number of factors have been associated with AAD/DAD in the general population. Few studies have explored these factors in DAD prisoners and most were again limited to incoming/remanded prisoners. Among the significant factors are: age, ethnicity [17], childhood conduct disorders, childhood abuse, school difficulties [17,19-22], additional substance-related disorder, previous psychiatric treatment, co-morbid psychiatric diagnosis, psychosis, mood disorder, antisocial personality, self-harm, suicide risk, history of serious illness or injury [16,17,21-23], fewer qualifications, unemployment, housing difficulties [21] and the length of time spent in prison [17]. AAD in prison has been generally ignored, yet alcohol has been widely related to violent behaviour and criminality. Alcohol consumption is forbidden in prison but alcohol trafficking and "home brew" production have been reported inside prisons. No studies have differentiated the profiles of AAD/DAD. Yet better knowledge of these differences could allow better screening as well as tailoring of specific prevention/treatment programmes.

The aim of this paper is 1/to assess AAD and DAD prevalence in a national randomized cross-sectional survey concerning the adult prisoner population, not restricted to incoming or remanded prisoners alone, assuming that a substantial proportion of prisoners continue or initiate substance misuse while in prison, 2/to compare AAD and AAD prisoners characteristics.

## Results

### Cohort description

The demographic profile of the population is presented in table 1. Four prisoners out of five were under fifty (mean age in years: 39 ± 13), and just above 80% had no, or very low educational achievement. Childhood history of adverse events was frequent and one quarter of the prisoners had had a contact with a children's judge before the age of 18 (Table 1). 46.6% (n = 465) had been already incarcerated and the most frequent crimes committed were against persons (52.7%) (Table 2). The mean time already spent in prison was 16.5 (SD = 22.3) months.

**Table 1:** comparison of socio-demographic status and child history variables for prisoners with a drug abuse/dependence (N = 278) or an alcohol abuse/dependence (N = 174)

		**Total**	**Drug abuse and dependence (last 12 months)**			**Alcohol abuse and dependence (last 12 months)**		
		**N = 998**	**Yes (278)**	**No (720)**		**Yes (174)**	**No (824)**	

		**%(N)**	**%**	**%**	**Odd ratio [IC95]**^1^	**%**	**%**	**Odd ratio [IC95]**^1^

**Socio-demographic status**								
*Gender*	Female	9.9(99)	6.5	11.2		7.1	10.6	
*Class of age (qualitative ordinate)*								
	18–29 years (ref)	29.0(289)	48.2	21.5	**1**	44.0	25.5	
	30–39 years	27.3(273)	23.3	38.1	**0.5(0.4–0.6)‡**	31.0	26.6	
	40–49 years	23.6(236)	28.3	11.4	**0.3(0.2–0.4)‡**	17.9	24.9	
	50–59 years	12.1(121)	2.2	16.0	**0.0(0.0–0.1)‡**	6.0	13.5	
	60 years and more	7.9(79)	0.0	11.0	**0.0(0.0–0.1)‡**	1.1	9.5	
*Education*	High school diploma (ref)	11.6(116)	7.9	13.1		4.9	13.1	1
	No education	45.7(455)	50.4	43.7		50.6	44.5	2.1(0.97–4.7)
	GCSE	35.3(352)	39.2	33.7		41.8	33.8	2.6(1.2–5.7)*
	University	7.3(75)	2.5	9.5		2.7	8.6	0.9(0.3–3.2)
*Marital status*	Single (ref)	41.2(411)	62.2	33.5	**1**	52.2	38.7	
	Married	36.0(358)	25.2	40.0	**0.4(0.3–0.6)‡**	29.3	37.4	
	Separated	19.7(196)	11.9	23.1	0.7(0.4–1.2)	17.4	20.5	
	Widow	3.0(30)	0.7	3.9	0.6(0.1–3.9)	1.1	3.4	
*Employment for at least two year *(yes)		63.2(574)	59.0	34.2		44.0	62.3	
**Child history**^§^								
	Judge in childhood	26.9269)	45.7	19.7		42.9	23.3	
	Separation from one parent > 6 month	43.2(431)	57.5	37.6	1.6(1.1–2.3)*	49.5	41.8	
	Family with a history of imprisonment	30.2(301)	41.7	25.7		40.2	27.9	
	Placement	21.9(219)	33.8	17.4		30.4	20.0	
	Death of a family member in childhood	35.1(350)	40.6	32.9		38.0	34.4	
	Ill-Treatment	29.8(297)	40.3	25.7		40.8	27.3	
	Previous history of trauma (other than ill-Treatment)	24.5(245)	34.2	20.8	1.9(1.3–2.9)**	29.9	23.3	

^1^The odd ratio presented were obtained after a logistic regression using a stepwise selection process (α = 0.05), adjusted on DSM IV psychiatric disorders, psychiatric history, temperaments, socio-demographic status, childhood history And imprisonment characteristics.

^§^"No" is the reference for all these variables

*p < 0.05 ** p < 0.01 † p < 0.001 ‡ p < 0.0001

**Table 2:** comparison of imprisonment characteristics for prisoners with a drug abuse/dependence (N = 278) or an alcohol abuse/dependence (N = 174)

		**Total**	**Drug abuse and dependence (last 12 months)**			**Alcohol abuse and dependence (last 12 months)**		
		**N = 998**	**Yes (278)**	**No (720)**		**Yes (174)**	**No (824)**	

		**%(N)**	**%**	**(N)**	**Odd ratio [IC95%]**^1^	**(N)**	**(N)**	**Odd ratio [IC95%]**^1^

*Type of prison*	"Center de detention" (ref)	25.0(249)	16.2	28.3	1	14.1	27.4	**1**
	"Arrest house"	55.0(549)	62.6	52.1	1.5(0.9–2.5)	72.3	51.1	2.0(1.2–3.4)**
	"Central house"	10.0(100)	4.0	12.4	0.7(0.3–1.5)	0.6	12.2	0.1(0.02–0.97)*
	"Overseas department"	10.0(100)	17.2	7.2	**5.7(2.9–11.3)‡**	13.0	9.3	1.7(0.8–3.4)
*Previous history of imprisonment *(ref: no)		46.6(465)	66.5	38.9	**2.6(1.8–3.9)‡**	59.2	43.7	
*Length of time spent in prison*	<= 6 months (ref)	33.3(332)	47.1	27.9		52.7	28.9	
	Six months-one year	25.7(257)	24.9	26.1		25.0	25.9	
	One year – two years	19.9(199)	16.5	21.2		14.7	21.1	
	Two years – five years	16.8(168)	10.8	19.2		7.0	19.1	
	Five years and more	4.2(42)	0.7	5.6		0.6	5.0	
*Length of sentence*	Less than six month (ref)	4.0(40)	7.2	2.8		8.1	3.1	1
	Between 6 months to one year	11.7(111)	21.9	7.8		23.9	9.0	0.9(0.4–2.0)
	Between one to five years	37.1(370)	47.2	33.2		49.5	34.2	0.7(0.4–1.7)
	More than five years	47.2(471)	23.7	56.2		18.6	53.7	**0.3(0.1–0.6)‡**
*Type of crime*	Crime against people (ref)	52.7(526)	33.1	60.3	**1**	45.7	54.3	**1**
	Crime against property	37.9(378)	54.0	31.6	**2.7(1.8–4.0)‡**	45.1	36.2	**0.5(0.4–0.9)†**
	Both	9.4(94)	12.9	8.1	1.7(0.9–3.1)	9.2	9.5	0.7(0.3–1.4)

^1^The odd ratio presented were obtained after a logistic regression using a stepwise selection process (α = 0.05), adjusted on DSM IV psychiatric disorders, psychiatric history, temperaments, socio-demographic status, childhood history and imprisonment characteristics.

*p < 0.05 ** p < 0.01 †p < 0.001 ‡p < 0.0001

The most frequent psychiatric diagnoses were current and lifetime mood disorders and current anxiety disorders, with nearly half the prisoners having one of these diagnoses. More than a quarter of the prisoners had attempted suicide and just under fifty percent had had a contact with a psychiatric department (Table 3).

**Table 3:** comparison of diagnosis and psychopathology variables for prisoners with a drug abuse/dependence (N = 278) or an alcohol abuse/dependence (N = 174)

		**Total**	**Drug abuse and dependence (last 12 months)-**			**Alcohol abuse and dependence (last 12 months)**		
		**N = 998**	**Yes (278)**	**No (720)**		**Yes (174)**	**No(824)**	

		**%(N)**	**%**	**%**	**Odd ratio [IC95%]**^1^	**%**	**%**	**Odd ratio [IC95%]**^1^

**DSMIV diagnosis**								
	Mood disorder (LT)	55.6(555)	67.6	51.0		71.2	52.1	
	Mood disorder(Ct)	45.6(455)	54.3	42.2		61.4	42.0	**2.0(1.4–3.0)†**
	Anxiety disorder(LT)	10.5(105)	13.3	9.4		14.6	9.5	
	Anxiety disorder(C)	54.5(544)	61.5	51.8		58.7	53.5	
	Psychotic disorder (LT)	24.7(247)	25.2	24.6		24.4	24.8	
								
	Psychotic disorder (C)	22.7(227)	22.3	22.9		22.8	22.7	
	Antisocial personality	31.5(315)	54.3	22.8		55.4	26.1	**2.3(1.5–3.4)‡**
	Alcohol abuse and dependence (12 m)	18.4(184)	39.9	10.1	**3.9(2.5–5.9)‡**	-	**-**	**-**
	Drug abuse and dependence (12 m)	27.8(278)	-	-	-	60.3	20.5	**3.5(2.3–5.3)‡**
**Psychiatric history**								
	Suicidal risk	40.5(404)	46.7	38.0		50.0	38.3	
	History of suicide attempt	28.7(287)	34.2	26.7		39.7	26.3	
	Psychiatric hospitalisation	16.2(162)	22.3	13.9		27.2	13.7	
	Psychiatric ambulatory care	36.7(366)	49.3	31.1		53.3	32.9	
**Temperament and character Inventory**								
	Novelty seeking low	39.7(396)	24.4	45.6	**1**	21.7	43.7	
	moderate	22.4(224)	20.1	23.3	1.3(1.0–1.6)*	22.8	22.4	
	high	37.9(378)	55.5	31.1	1.6(1.3–2.0)*	55.5	33.9	
	Harm avoidance low	44.5(444)	47.1	43.5		41.3	45.2	
	moderate	25.1(251)	19.1	27.5		22.3	25.8	
	high	30.4(303)	33.8	29.0		36.4	29.0	
	Reward dependence low	25.4(254)	27.3	24.7		26.6	25.2	**1**
	moderate	31.7(316)	25.2	34.2		34.3	31.1	0.7(0.6–0.9)*
	high	42.9(428)	47.5	41.1		39.1	43.7	0.6(0.2–0.8)*
	Low persistence	20.0(200)	27.0	17.3		29.3	17.9	
	Low cooperativeness	18.0(180)	25.5	15.1		25.5	16.3	
	Low self-directedness	29.8(298)	47.8	22.9	**2.1(1.4–3.1)†**	44.6	26.5	
	Low self-transcendence	37.6(375)	51.1	32.4		47.3	35.4	

LT : lifetime C: current

^1 ^The odd ratio presented were obtained after a logistic regression using a stepwise selection process (α = 0.05), adjusted on DSM IV psychiatric disorders, psychiatric history, temperaments, socio-demographic status, childhood history and imprisonment characteristics.

*p < 0.05 ** p < 0.01 †p < 0.001 ‡p < 0.0001

### Prevalence of drug and alcohol abuse/dependence in prison (table 1)

35.2% of prisoners presented either AAD or DAD in the last 12 months. 18.4% had presented AAD and 27.9% DAD in the last 12 months. 11.2% (N = 111) had both diagnoses in the previous 12 months.

Cannabis was the most frequently used drug in the previous 12 months (26.7%), others drug use being marginal (2.7% for opiate to 5.4% for cocaine/crack).

### Socio-demographics and childhood history of prisoners with AAD/DAD (table 1)

Sociodemographic factors associated with DAD were very different from those associated with AAD (table 1).

Married prisoners were less likely diagnosed DAD than non-married (OR = 0.4), as well as the older prisoners compared to the younger (OR = 0.5), and finally those separated early in childhood from one parent were at higher risk (OR = 1.6).

Compared to non-users, prisoners with an AAD had a lower level of education (significant for low-grade technical diplomas (OR = 2.6)).

### Imprisonment factors associated with alcohol and drug abuse/dependence in prison (table 2)

DAD and AAD prisoners were also different for this set of variables (table 2).

Prisoners with DAD were over-represented in the overseas *département *(OR = 5.7), and among re-offenders (OR = 2.6). They had more frequently committed crimes against persons (OR = 1.9, ref: crimes against property), and were more often subjected to disciplinary measures (OR = 1.7).

Prisoners with AAD were over-represented in *maisons d'arrêt *(short term) and under-represented in *prisons centrales *(long term), less likely to be sentenced to more than five years (OR = 0.26) and had more frequently committed crime against property (OR = 2.7).

### Psychiatric disorders and temperament of prisoners with drug or alcohol abuse/dependence (table 3)

AAD and DAD were reciprocally associated (OR > 3) but their psychiatric and TCI profiles were clearly different (table 3).

DAD was not associated with another psychiatric disorder but was more frequent in case of marked novelty-seeking (OR = 1.2) and low self-directedness (OR= 2.1)

AAD was significantly associated with current mood disorder (OR = 2.0), antisocial personality (OR = 2.3) and low reward dependence

## Discussion

The data from this random national sample of 998 French adult prisoners in 23 prisons, including short and long term sentences, highlights the following:

1. More than a third of prisoners had either an AAD or DAD in the previous 12 months. Cannabis was the most frequent drug used.

2. While drug use in prison is a well-known problem, alcohol use also seems frequent, with nearly a fifth of prisoners presenting AAD.

3. Finally, while no direct comparisons were performed, risk factors associated to ADD and risk factors associated to DAD were distributed differently. These two groups also differed from other prisoners regarding some of these variables

### Strengths and limits of the study

One strong point of this national random study is that all types of prisoners were assessed, and not only prisoners on remand or incoming prisoners, and that it involved the different types of French prison settings (which vary considerably in terms of security, length of sentence, etc.). We can thus assume that our sample is representative of French prisoners, and that it can give an overview of drug use during imprisonment. It is however possible that prisoners incarcerated for more than 12 months, compared to incoming prisoners, under-reported their past substance use, as it necessarily took place in prison, fearing sanctions in case of breach of confidentiality. However, the high prevalences found are not in favour of under-reporting.

A report bias may have occurred for prisoners incarcerated less than 6 months, as their substance use may have been interrupted in jail. The link with "maison d'arret" (where most of the sentences are short term) should then be taken cautiously.

Another point is that no objective measures (urine/blood tests) of substance use were performed and that the diagnoses were based only on a clinical assessment. This is a general problem encountered in substance-use disorder (SUD) prevalence studies, but this tendency to under-report SUD may be greater in prison. Indeed, prisoners may fear disciplinary measures or reduced access to their supplier if they report substance use. Moreover, most of the standardized diagnosis instruments are not validated in prison, where antisocial personality diagnosis is frequent, and where prisoners may falsify report on their psychiatric status to gain benefits from "patient" status. We limited the effect of the setting by 1) ensuring strict confidentiality of the interview 2) using a diagnosis assessment procedure that improved inter-rater agreement 3) adjusting results on the presence of antisocial personality. It should be noted that blood/urine tests alone may not be more valid than a good clinical assessment, since for drugs with a short lifetime, false negative results could be frequent and repeated measures too costly.

### Prevalence of alcohol and drug abuse or dependence in prison

The first point to be made is that psychiatric disorder prevalence rates found in this study are higher than most of those already published. One of the main discrepancies concerns current and lifetime mood disorders (affecting around half the prisoners) while a systematic review of surveys in general prison populations in western countries shows prevalences not exceeding 10% [24]. Several non-exclusive explanations may be proposed for these differences: first, these studies used different instruments; second, none was conducted in France (in favour of this, a paper published in 2004 [25] reports that prevalence of depression was as high as 27.4% in French prisons); third, certain depressive symptoms are over-represented because of the prison context (e.g. sleeping disorders).

The second point, often neglected in studies, is that AAD prevalence observed here also very high. This could be, as in Finland and Denmark, the effect of national culture, but an alternative explanation is that only illicit drugs are generally investigated.

Thirdly, cannabis was the most frequent drug used, concerning a little over one in four prisoners, five times more than opiate use. Previously, opiate dependence has been reported as the most frequent drug disorder in prison [9]. This new trend reflects current French general population use, where cannabis is widely used and where opiate use and its crime-related consequences have decreased with the introduction of maintenance treatment [5]. However, cannabis has also been trivialized and tolerated by prison authorities compared to other drugs which could thus have been under-reported.

Finally, certain socio-demographic, psychopathological and imprisonment characteristics clearly distinguish prisoners with DAD from prisoners with AAD. These two groups also differed from other prisoners regarding some of these variables.

### Comparison with the British national study of substance misuse in prison

When compared with the aforementioned British national study of substance misuse in prison [14-17], the conclusions are quite similar. Indeed, a very high prevalence of substance misuse (42.5%) were drug dependent and alcohol problem (close to 30% had a severe alcohol problem according to the AUDIT). Another interesting point is that cannabis is also the first drug reported in both study with similar prevalence (around 60%). However, while other drugs are rarely reported in our study (cocaine being the most frequent with 5.7% of prisoners reporting use), other drugs were relatively frequently used in British prisons (between 19.1% (for cocaine) to 32.6% for stimulants). They may be different explanations which are not mutually exclusive: a fear of confidentiality breach, a higher general prevalence of cocaine and stimulant use in Great Britain, a difference in the availability of opiate maintenance treatment in the two countries, etc. However, comparing this two study remains difficult as the British study mainly assess SUD prior to prison sentence while our assessment focus on the last 12 months prevalence, aggregating both prior and during prison sentence term use. Our dependent variable is quite different too, as we it covers both abuse and dependence. However, we can conclude that in both countries substance use disorder is overwhelmingly represented in prison compared to the general population.

### Comparison with the general population

When compared to the available national data on drug use in the French general population, our data are unequivocal and show that addicts are overwhelmingly overrepresented in prisons: 2.8% of French male adult have a regular use of cannabis (but 18% of 17–18 years old have a problematic use), less than one percent have ever used heroin and 0.6% used once cocaine in the last 12 months among the 15–64 [26]. When compared to our variable "first drug used" (which is not strictly correct, but give an idea of the discrepancy), the ratio is close to 9 times higher in prison for cocaine and cannabis.

### Psychopathology: comorbid psychiatric disorders

Concerning psychiatric co-morbidity, only prisoners with an AAD seem to have greater vulnerability, restricted to current mood disorder, an already well-established association in general population [27]. We did not find any relation between psychosis and drug use, as reported by recent British studies [15], but these results were contradictory in one study [16] depending on the type of drug used. It is particularly true for cannabis, that was the most frequent drug reported in our study and which has been moderately but significantly linked to psychosis in different studies in general population (for a review of this question, see [28]). One explanation is that our group was more heterogeneous, aggregating abuse and dependence and thus probably less severely dependant than the group studied in this British study. Other differences in assessing the primary variable may explain this discrepancy between the two studies (see above). Additionally, our dependant variable, which covers a broad range of substance abuse problem, has the inconvenient that it may hide some associations with psychiatric comorbidities or temperaments, which could be restricted to the most severely dependent individuals. However, associations with these more severe groups have already been reported in other studies and we provide an approach that emphasizes a broader public health approach, as not only dependence but abuse has been associated to psychological difficulties. Furthermore, the causal impact of cannabis may be difficult to highlight in prison, as prisoners with or without addiction are submitted to other strong influences potentially responsible for psychosis (sensory isolation, aggressive environment, etc.) that can not be objectively assessed and play the role of major confounding factors.

Concerning anxious and mood disorders, while cannabis has been involved in their development, the level of evidence is not as important as for alcohol (for review see [29]) and no consensus has been reached on this topic. It is thus not surprising that no association is found for the DAD group with these disorders.

Antisocial personality is classically expected to be more frequent in DAD than in AAD, in line with the social representation of deviance associated with drugs, alcohol being legal while drugs are not. Contrary to our expectations, antisocial personality was significantly linked to AAD but not to DAD. Two explanations can be proposed: 1) the relationship between antisocial personality and DAD may be restricted to opiate users. Indeed, in our study, cannabis superseded other drugs. Though cannabis is not legal in France, it is not tagged with the same social taboo as opiates. 2) Prisoners with an AAD seem to correspond to a certain type of alcoholism, more prone to violent crime and less reward-sensitive, as we will detail below.

### Psychopathology: temperament and character

Some personality features have been commonly linked to patients with SUD, the most salient variables being novelty-seeking [30,31], impulsivity, and low harm avoidance [32,33]. Few studies have tried to differentiate between drug preferences (voir réf 36) and none have studied this among jailed substance users. Results are contradictory but some papers have reported clear differences: healthy subjects with less attachment (a subdimension of reward dependence) were more ethanol-preferring than benzodiazepine preferring [34]; the constraint dimension of Tellegen's Multiple test discriminated between AAD and DAD [35]. A recent study using TCI reported that heroin users scored higher than subject with an alcohol-related disorder on novelty-seeking and self-directedness, and that exploratory excitability (a sub-dimension of novelty-seeking) was the best variable to segregate these groups. Subject with an alcohol dependence also had a lower score than controls for certain subscales of reward dependence [32]. Our study confirms some of these results. However, novelty-seeking does not seem to be the best discriminating factor, having a lower odds ratio compared to low self-directness for DAD and low reward dependence for AAD. These data may be useful to discriminate these populations, but they are also interesting for the tailoring and allocation of specific rehabilitation programs. Indeed, drug users have low self-directedness and are therefore considered to be more impulsive, to have riskier and more restricted modes of self-stimulation (through novelty-seeking) but they are not prone to social withdrawal. Conversely, prisoners with an AAD have a low reward dependence temperament, which means they are socially insensitive, irresolute and indifferent if alone, which leads to social withdrawal.

Overall, jailed individuals with an AAD seem to differ more from individuals with an AAD in the general population (for whom novelty-seeking is the most sensitive factor [33,36]), than jailed drug users differ from drug users in the general population. Indeed, prisoners with an AAD have a low reward dependence temperament, are more prone to crime against persons, have lower educational status and a more antisocial personality. This is very close to the description of Cloninger's alcoholic subtype II [37], which is believed to be more genetically loaded and associated with antisocial behaviours.

### Childhood history

Prisoners with DAD had suffered more frequently from childhood separation and trauma other than ill-treatment. These associations have been widely reported in the literature (for example [38,39]) but rarely demonstrated. Separation from parents could lead to an insecure attachment style which is associated with antisocial behaviours. It should be noted that insecure attachment style was more frequent among prisoners in a recent study [40]. For alcohol-related disorders, in contrast, a recent longitudinal study confirmed the lack of relationship between childhood factors and adult drinking [41] while family seems to be a more protective factor for DAD than for AAD [42]. For trauma other than ill-treatment, subjectivity is known to be a major bias in traumatic stressor report, in particular in SUD [43], and interpretation should be cautious.

### Imprisonment characteristics

Imprisonment characteristics were also different. One unexpected finding is that in the overseas département prison being imprisoned is strongly correlated with DAD. This could be explained by laxer security and by the geographical situation of overseas départements, making them a privileged channel for drug flow. Another point is that re-offending is associated with drug abuse/dependence. This was expected, as drug consumption is an illegal act that can per se lead to prison. The fact that crime against persons is more associated with alcoholism and crime against property with drugs is not a surprise. One direct explanation is that crime against persons is the consequence of alcohol loss of inhibition for aggressive behaviour, while prisoners with DAD commit crime to obtain their drug.

### Public health and policy implications

Money and human resources allocated to mental health programmes in prison are very limited in most European countries. Can these profiles distinguishing between jailed AAD and DAD subjects be exploited to tailor more specific prevention/treatment/rehabilitation programs?

According to our results, which need to be replicated, programmes focusing mainly on addiction could be applied to prisoners with a drug-related disorder, while additional psychiatric care or integrated programmes should be favoured for prisoners with an alcohol-related disorder. Prisoners with an AAD also seem to be in greater need of education and social skills, while prisoners with a DAD should be directed toward programmes stressing other means of stimulation than novelty-seeking and focusing low self-directedness (anger management program, problem-solving skills, etc.). However, compared to self-directedness which is believed to be a learned trait that thus may be improved by therapy both novelty-seeking and reward-dependence are temperament-based, more genetically loaded and less sensitive to learning. Furthermore, caution is required in translating these results into practice, as no longitudinal data has proven the efficiency of preventive strategies based on personality screening, which could moreover stigmatize extreme personalities.

## Conclusion

This study shows the importance of regular screening of all prisoners and not only incoming prisoners, if we want to address the drug use/initiation problem in prison.

Finally, a crucial public health question is the high prevalence of SUD observed in prison, with cumulating evidence in the literature of maintenance or initiation while in prison: Are prisons the right places to direct these people, many of whom have committed offences in relation drug legislation? Consideration of these issues by addiction specialists, as well as judiciary and prison specialists, is required to develop alternative settings to prison for these people.

## Methods

### Study design

In the year 2002, the French ministries of health and justice decided to make prevalence estimates for mental disorders in French prisons. A cross sectional study of 1000 prisoners selected from 22 prisons using a stratified random sample strategy was conducted between September 2003 and July 2004.

### Selection of prisons

There are three types of prison in France. "Maisons d'Arrêt" are intended for remand prisoners or prisoners with short sentences. "Prisons centrales" are for prisoners with long sentences entailing maximum security. "Centres de détention" are intermediate.

### Selection of prisoners

A number of 1000 prisoners to recruit was calculated to detect a pathology with a prevalence of 20% with a 95% confidence interval of ± 2.5%. 998 subjects were indeed sampled from these prisons between September 2003 and July 2004: 100 women, 100 men from France's overseas départements and 798 men from metropolitan France. This number of subject was chosen 1) to allow a good feasibility according to the budget of the study 2) to avoid a design effect in the calculation of statistical power. The 998 prisoners were selected using a stratified random sampling strategy. 23 prisons centres were randomised among the 188 prisons existing in France at the time of the study: 13 maisons d'arrêt (two having a capacity of more than a 1000 prisoners, 3 between 400 and 1000, 4 between 100 and 400, and 4 less than 100), 5 centres de detention (2 national and 3 regional centers), 2 prisons centrales, one male centre in a French overseas département and two prisons for women were chosen at random (for women, no prison centrale was selected). Prisoners were then selected at random in the population of each selected prison. The percent of prisoners selected in each prison was between 1.4% (for "maison d'arret") and 5.1% (for "maison centrale") according to the real size of each center, which correspond to the recruitment of 50 prisoners by center (except for the 4 centers of less than 400 prisoners were the number was 37 prisoners and 13 prisoners for the 4 centers of less than 100)."

After fully describing the study to the subjects, written informed consent was obtained.

### Data collection, diagnosis procedure

Each prisoner was interviewed for approximately 2 hours by a group of 2 clinicians (clinical psychologist or psychiatrist). At least one of these clinicians had to be a senior psychiatrist; neither could belong to the prison medical team.

Diagnoses were then collected according to a semi-structured procedure validated in a previous study [44]: one of the clinicians used a structured clinical interview generating DSM IV diagnoses (MINI plus v 5.0 [45]); the second, more experienced, completed the procedure with an open clinical interview, intended to be more clinically relevant. The interview continued with the clinician-version of Cloninger's Temperament and Character Inventory (TCI) [46] and various questions on socio-demographics, legal status, previous treatment and history of trauma. At the end of the interview, each clinician independently summarized his/her list of diagnoses [47]; then they met and agreed on list of diagnoses.

All interviewers had specific training in the methodology and the instruments used in this study.

### Sample validation

57% of prisoners were available at the time of interview and accepted. Collaboration with prisoners was good to very good 88% of the time and average 10% of the time. There was no problem speaking/understanding French in 85% of cases.

Inter-rater agreement on diagnosis assessment for the total sample was estimated. All the values of Cohen's Kappa corresponded to "good" (> 0.6) or "excellent" (> 0.8) agreement [48], with very high scores for alcohol dependence (0.91), and drug dependence (0.95). Only hypomania/mania had moderate inter-rater agreement (0.53).

### DSM-IV psychiatric diagnoses

The 23 possible diagnoses were grouped into 9 diagnoses, recorded as current (C) and/or lifetime (LT): mood disorders (major depressive disorder (C/LT), dysthymia (C), mania/hypomania (C/LT), bipolar disorder (LT), psychotic symptoms limited to depressive/manic episode (C/LT)), anxiety disorders (panic disorder (C/LT), agoraphobia (LT), generalized anxiety (LT), social phobia (LT), obsessive-compulsive disorder (LT), post-traumatic stress (LT)), psychotic syndrome (schizophrenia (CT/LT), brief psychotic disorder (C/LT), schizophreniform disorder(C/LT), schizoaffective disorder (C/LT), delusional disorder (C/LT), psychotic disorder not otherwise specified (C/LT) and antisocial personality (LT). Prisoners with either a drug abuse or dependence disorder consensual diagnosis in the last 12 months were labelled DAD (Drug abuse or dependence), while those with either a alcohol abuse or dependence disorder in the last 12 months were libelled AAD (alcohol use or dependence). Those two variables are thus the aggregation of both prisoners with an abuse or dependence disorder in the last 12 months, respectively for alcohol and drug. They were design to cover a broad range of severity of substance use, in term of regular use or adverse consequences.

### Ethics

This study was considered as having a direct individual benefit according to CCPPRB (French ethical committee) decision since a procedure to signal prisoners to the prison medical team was provided for in two cases: in case of psychiatric emergency (particularly high risk of suicide), with or without the permission of the patient, and in cases where psychiatric disorders were deemed serious, only with the permission of the prisoners. A form was designed for this procedure. About 22% of prisoners were signalled to the prison medical team (with the agreement of the prisoners; patients already followed for psychiatric reasons were not signalled).

### Statistical analysis

Statistical analyses were performed with SAS 8.6, except the multiple imputation of missing data, which was performed with the Gibbs sampler package of R 2.0.1 [49]. Two tailed T-test and Pearson CHI-2 test were used for the univariate analysis. Multiple logistic regressions using a stepwise selection process were used to analyse the factors associated with 1) drug abuse/dependence disorder in the past 12 months 2) alcohol abuse/dependence in the past 12 months. The fit of the model was good and convergence criteria were met.

A multiple imputation procedure was used to take account of missing data. They were no missing data for consensual diagnosis. Only the following variables had more than 2% of missing data: type of crime committed (5.6%), work in prison (3.1%), length of sentence (27.25%), and TCI variables (11.12%). n 2% of missing data except for type of crime committed (5.6%), work in prison (3.1%), length of sentence (27.25%), and TCI variables (11.12%). No major differences were observed between the multiple logistic regressions with or without imputations.

## Competing interests

The author(s) declare that they have no competing interests.

## Authors' contributions

M.L participated to the design, the statistic and the draft of this paper

B.F participated to the design and the statistic of this paper

I.G participated to the design and the draft of this paper

X.N participated to secondary statistical analysis

L.M criticaly revised this paper and gave expert opinion

M.R criticaly revised this paper and gave expert opinion
